# Correspondence between primary and secondary care about patients with cancer: a Delphi consensus study

**DOI:** 10.1007/s00520-019-04712-5

**Published:** 2019-03-01

**Authors:** M. E. Stegmann, T. M. Homburg, J. M. Meijer, J. Nuver, K. Havenga, T. J. N. Hiltermann, J. H. Maduro, J. Schuling, D. Brandenbarg, A. J. Berendsen

**Affiliations:** 1 University Medical Center Groningen, Department of General Practice and Elderly Care Medicine, University of Groningen, HPC FA 21, Postbus 30.001, 9700 RB Groningen, The Netherlands; 2 University Medical Center Groningen, Department of Medical Oncology, University of Groningen, Groningen, The Netherlands; 3 University Medical Center Groningen, Department of Surgery, University of Groningen, Groningen, The Netherlands; 4 University Medical Center Groningen, Department of Pulmonary Diseases, University of Groningen, Groningen, The Netherlands; 5 University Medical Center Groningen, Department of Radiation Oncology, University of Groningen, Groningen, The Netherlands

**Keywords:** Correspondence, Oncology, Primary care, Secondary care

## Abstract

**Introduction:**

To provide optimal care for patients with cancer, timely and efficient communication between healthcare providers is essential. In this study, we aimed to achieve consensus regarding the desired content of communication between general practitioners (GPs) and oncology specialists before and during the initial treatment of cancer.

**Methods:**

In a two-round Delphi procedure, three expert panels reviewed items recommended for inclusion on referral and specialist letters.

**Results:**

The three panels comprised 39 GPs (42%), 42 oncology specialists (41%) (i.e. oncologists, radiotherapists, urologists and surgeons) and 18 patients or patient representatives (69%). Final agreement was by consensus, with 12 and 35 items included in the GP referral and the specialist letters, respectively. The key requirements of GP referral letters were that they should be limited to medical facts, a short summary of symptoms and abnormal findings, and the reason for referral. There was a similar requirement for letters from specialists to include these same medical facts, but detailed information was also required about the diagnosis, treatment options and chosen treatment. After two rounds, the overall content validity index (CVI) for both letters was 71%, indicating that a third round was not necessary.

**Discussion:**

This is the first study to differentiate between essential and redundant information in GP referral and specialist letters, and the findings could be used to improve communication between primary and secondary care.

**Electronic supplementary material:**

The online version of this article (10.1007/s00520-019-04712-5) contains supplementary material, which is available to authorized users.

## Introduction

The incidence of cancer is rising in Europe [1, 2]. Cancer care is complex in most instances, requiring the involvement of multiple healthcare providers for diagnosis and treatment [3–5]. Consequently, a patient may visit many different healthcare providers over a relatively short and intense period.

In the Netherlands and other countries with a similar system, all inhabitants are registered with a general practitioner (GP). This GP functions as gatekeeper and coordinator of healthcare. In countries with such a system, patients often consult their GP for support [6, 7]. To provide this care in an optimal manner, timely and efficient information exchange is essential, both at time of diagnosis and during therapy [5, 8, 9]. As the electronic patient records of GPs and medical specialists are separate, the most common way of communication is the digital exchange of letters between GP (i.e. referral letters) and specialists (i.e. consultation and treatment) [10]. These must be relevant and complete, particularly with regard to the specific needs of the recipient party. Inadequate correspondence by GPs may lead to unnecessary repetition of diagnostic tests, patient anxiety and low patient satisfaction [4, 11]. Timely and relevant information from specialists can enable GPs to support their patient to make treatment decisions, give moral support during treatment and monitor and treat side-effects and complications [10]. Despite all parties agreeing that adequate correspondence is important, it is equally agreed that the current situation could be improved [8, 12].

We aimed to find consensus among GPs, oncology specialists (i.e. oncologists, radiotherapists, urologists and surgeons) and patients regarding the content of referral and specialist letters before and during the initial treatment (i.e. until start of treatment as described in the original treatment plan) of cancer.

## Methods

### Study design

We used the Delphi procedure to reach consensus about the required content [13]. According to our institutional review board, no approval was needed because this study was not subject to the Dutch Medical Research Involving Human Subjects Act. All participants were informed about the aims of the study and agreed to participate.

### Composition of the item list

The initial list of items was based on two sources: the code list of an earlier qualitative study [14] and the results of a literature search. The literature search was performed in Medline and Web of Science (Supplement 1, Fig. S1a). The items derived from the qualitative study and the literature search were discussed until consensus on every item was reached among four members of the research group (MH, MS, JS and AB). To test the applicability, the item list was presented to two GPs and two medical specialists in individual sessions.

### Composition of the expert panels

The following three expert panels were formed by purposive sampling to optimise diversity: a GP panel, an oncology specialist panel (i.e. oncologists, radiotherapists, urologists and surgeons) and a patient panel (patients or close relatives). Potential participants were invited from different fields, ensuring variety in characteristics such as age, gender, academic qualification and place of employment. Patient associations in the Netherlands were approached to identify patients. Given the number of sample characteristics, we aimed to include 20 GPs, 20 oncology specialists and 15 patients after the final round.

### Data collection and analysis

The Delphi procedure was performed over at least two rounds, with participants using the online Qualtrics tool, version 2005, to complete questionnaires (Qualitrics, Provo, Utah, USA). The patient panel received a list of definitions for the items used in the questionnaire. During round one, we also sent a short questionnaire about demographic characteristics and preferences for the layout and writing style of the respective letters. Descriptive statistics were applied to the answers of these questions. All data were analysed using IBM SPSS for Windows, version 22.0 (IBM Corp., Armonk, NY, USA).

### Delphi procedure

#### Round 1

Participants were asked to rate each list item on four-point Likert-type scales (not relevant = 1 point, moderately relevant = 2 points, relevant = 3 points and highly relevant = 4 points), with a fifth “no opinion” option (0 points). Participants were provided with a free text area at the end of the questionnaire in which they could propose additional items. The median scores for each item were calculated for the panels combined and for each panel individually. If the difference between the median scores of the GPs and the specialists was ≥ 2 points, the item score of the receiving party was weighed more heavily (weight factor 1:2). Items with median scores < 2.5 were considered not relevant. Items with median scores ≥ 2.5 were included in the concept consensus list: those with scores ≥ 2.5 but < 3.5 were considered less relevant and those with scores ≥ 3.5 were defined as highly relevant. Additional items were added to the concept consensus list if more than one participant suggested the same item.

#### Round 2

Participants were asked to indicate whether they agreed (yes/no) with inclusion of the highly relevant items on the consensus list. Based on the content validity index (CVI, which is the proportion of respondents who agree with the proposed relevance of the item), if ≥ 70% of respondents disagreed with a proposed item, it was excluded from the final list [13, 15]. For all items scored as less relevant in the first round, participants were asked to indicate whether they agreed (yes/no) with their exclusion from the consensus list; if ≥ 70% of respondents disagreed with this proposal, the item was included in the final consensus list [12, 13]. Participants were not asked to review items with low relevance a second time. If ≥ 70% of the items included in round two reached a CVI of ≥ 0.70, an additional (third) round was not deemed necessary. Agreement (CVI) between the panels was calculated as the percentage of items in which the different panels separately reached the same decision.

## Results

### Composition of the expert panels

In both rounds, GPs (*n* = 39, 42%), medical specialists (*n* = 42, 41%) and patient representatives (*n* = 18, 69%) completed the whole questionnaire or at least 70% of the questions. The respondents’ characteristics met the inclusion requirements (Table 1). Members of the panels were more mostly experienced clinicians and older patients.

**Table 1:** Panel characteristics

Characteristics	GPs (*n* = 39)	Medical specialists (*n* = 42)	Patients (*n* = 18)
Age (years)	Mean 50.5, SD 8.8	Mean 47.6, SD 7.9	Mean 64.8, SD 8.2
Male, *n* (%)	20 (51%)	28 (67%)	12 (67%)
Academic title MD–PhD, *n* (%)	11 (28%)	26 (62%)	
Involved in training GPs/medical specialists, *n* (%)	22 (56%)	25 (60%)	
Active/board member of (scientific) union/patient association *n* (%)	22 (56%)	20 (48%)	9 (50%)
Employed, *n* (%)			
City area	14 (36%)		
Semi-urban area	14 (36%)		
Rural area	11 (28%)		
University hospital		17 (40%)	
Leading general hospital		9 (21%)	
Peripheral hospital		16 (38%)	
Type of employment, *n* (%)			
Self-employed	34 (87%)	16 (38%)	
Paid employment	5 (13%)	26 (62%)	
Type of practice, *n* (%)			
Solo practice	5 (13%)		
Duo practice	12 (31%)		
Group practice	14 (36%)		
Family practice center	8 (21%)		
Discipline, *n* (%)			
Medical oncologist		11 (26%)	
Surgeon		7 (17%)	
Radiation oncologist		3 (7%)	
Pulmonologist		12 (29%)	
Urologist		2 (5%)	
Gynaecologist		2 (5%)	
Gastroenterologist		2 (5%)	
Other		3 (7%)	
Situation, *n* (%)			
Patient representatives			17 (94%)
Close contact of patient			1 (6%)
Type of cancer, *n* (%)			
Breast			2 (11%)
Lung			5 (28%)
Prostate			10 (56%)
Colorectal			1 (6%)

### Composition of the item list

The process for composing the original item list for panel review is shown in the supplementary material (Supplement 2, 7ure S1 b–c). In round one, the differences between the median scores of GPs and specialists were never ≥ 2 points, so no weighting was applied. In round two, the overall CVI for both letters was 71%, indicating that a third round was not necessary. For those items for which no agreement was reached, the patient panel was usually inclined to include the item, while the GPs and medical specialists were not.

### Delphi procedure: referral letters

For referral letter content, 72 items were included in the original list. The participants rated 12 items (17%) as highly relevant, 45 items (63%) as less relevant and 15 items (21%) as not relevant (Supplement 2). Thus, the consensus list for referral letters consisted of 57 items after the first round. All 12 highly relevant items from round one remained after round two, but consensus did not reach the required level for any of the 45 less relevant items. Therefore, only 12 items were included in the final consensus list for the referral letters (Table 2). These items mainly concerned medical facts (e.g. past medical history, resuscitation policy and medication details [names, doses and allergies]). Besides requiring the reason for referral and the level of urgency, information was also required about the presenting symptoms and the history of symptoms, as well as any aberrant findings on both physical examination and investigation. Regarding psychosocial information, the only retained item was the need for an interpreter when a language barrier was present. However, there was no requirement for a provisional diagnosis or for treatment, contextual or psychosocial information.

**Table 2:** Desired content of referral letter and specialist letter

Category	Referral letter	Specialist letter
Purpose	Reason for referral Level of urgency	Purpose of the letter
Medical facts	Active diagnoses/history Resuscitation policy Medication allergies Medication names Medication doses and frequencies	Active diagnoses/history Resuscitation policy Medication allergies Medication names Medication doses and frequencies Any clinical trial the patient is involved in*
History	Presenting symptoms History of symptoms	Presenting symptoms
Psychosocial information	Need for an interpreter	
Diagnostic pathway	Physical examination: Aberrant findings, relevant for current problem Investigations: aberrant findings, relevant for current problem	Physical examination: aberrant findings, relevant for current problem Investigations: aberrant findings, relevant for current problem
Diagnosis		Provisional diagnosis/diagnosis Whether tumour is localised of metastasized Description of size and direct expansion of primary tumour** Description of expansion to regional lymph nodes Description of distant metastasis
Treatment		Treatment options Selected treatment (e.g. watchful waiting or surgery) Explanations for chosen treatment Aim of treatment: curative or palliative Summary/conclusion of multidisciplinary consultation Prognosis with treatment Short-term side-effects of treatment* Effective initiated treatment Response on initiated treatment
Completion		Summary/conclusion What the patient has been told For what problems should the patient contact the medical specialist? Whom should the patient contact Specific request to the GP
Hospitalisation		Discharge destination Complications Medication changes Complete medication list Active medical problems at discharge Intentions regarding residual medical materials (e.g. drains or stitches)

*Less relevant in round 1

** Less relevant in round 1 and also overall score < 70% in round 2 (62.7%), but included because 71.4% for GPs in round 2

### Delphi procedure: specialist letters

Of the original 108 items, 32 (30%) were rated as highly relevant, 51 (47%) as less relevant and 25 (23%) as not relevant (Supplement 2). After the second round, all 32 highly relevant items remained on the list and 3 less relevant items were included. Therefore, the final consensus list for specialist letters contained 35 items (Table 2). Three new items were proposed in round one, but each was only suggested once (Supplement 2).

In the final consensus list for specialist letters, the requirements were for the purpose of the letter to be stated, for the corresponding medical facts to be included from the referral letters (e.g. history, resuscitation policy, and medication details) and for details of any trial enrolment. The requirement for information about the diagnostic process was limited to details about presenting symptoms, physical examination (abnormal findings relevant to the current problem) and investigation results (aberrant findings relevant to the current problem). Concerning the diagnosis, it was agreed that the following six items should be included: provisional diagnosis/diagnosis, whether the tumour is localised or metastasised, a description of the size and local invasion of the primary tumour, whether there is spread to regional lymph nodes, and whether there is distant metastasis. Concerning treatment, the following eight items were desired: the option(s), the selected option(s), the explanation(s) given, the aims (curative or palliative), a summary/conclusion of any multidisciplinary meeting, the prognosis, the short-term side-effects, the expected effectiveness, and the response to date.

There were also a few other requirements of the included information. Five completion-related items were included, such as the provision of a summary/conclusion, details of what the patient has been told and whom the patient should contact. When a patient was hospitalised, six additional items were requested, such as the discharge destination.

### Layout and writing preferences

All parties preferred referral letters to be short (mean 1.36 pages) and specialist letters to be longer (mean 1.76 pages). However, the panels did not agree about the preferred structure or writing style (Table 3). The oncologists preferred a structured format, unlike the other two panels, which preferred an unstructured format with no predefined sections. Moreover, most patient representatives thought the information could be best communicated in a short-hand or abbreviated medical style (e.g. “chemotherapy delayed because of cough, whereas half of the doctors was in favour of full phrases (e.g. “We delayed chemotherapy for one week because the patient developed a cough”). Most respondents thought that correspondence from specialists should not include all information available in the hospital records, and only a minority advocated using jargon (specialism-specific abbreviations, e.g. “hipec” for “hyperthermic intra peritoneal chemotherapy”). However, all parties agreed that it was appropriate to use abbreviations that are in common use (“e.g.” for exempli gratia) and most GPs thought that common medical abbreviations (e.g. BP for blood pressure) were appropriate (Table 3).

**Table 3:** Layout and writing preferences

Referral letters					Specialist letters			
	GPs (%)	MSs (%)	Ps (%)	Overall (%)	GPs (%)	MSs (%)	Ps (%)	Overall (%)
Format and writing style								
A structured format is important	54.0	61.8	61.1	58.5	43.2	71.8	66.6	59.6
I prefer phrases above short-hand or abbreviated medical style	45.9	46.2	22.2	41.8	54.1	64.1	22.2	52.1
The specialist letter should contain all information available in hospital records					25.6	35.7	72.2	38.4
Use of abbreviations								
No abbreviations	2.7	2.6	11.1	4.3	2.7	5.1	11.1	5.3
Only general abbreviations	35.1	46.2	77.8	47.9	35.1	46.2	72.2	46.8
Also medical abbreviations	54.1	38.5	5.6	38.3	59.5	35.9	11.1	40.4
Also jargon abbreviations	8.1	12.8	5.6	9.6	2.7	12.8	5.6	7.4

*GPs* general practitioners, *MSs* medical specialists, *Ps* patients

## Discussion

In the two-round Delphi process, GPs, medical specialists and patient representatives reached consensus on lists for the most relevant items in correspondence between primary and secondary care.

It was agreed that referral letters should contain medical facts and information about the current problem, but that a detailed description of the physical examination and investigation findings was not necessary. Information about social context, diagnosis and treatment to date was considered unnecessary. It was considered that the specialist letters should contain the same medical facts as the referral letters. Also, it was agreed that diagnosis and treatment should be described in detail, and that items such as “what the patient has been told” and “whom the patient should contact” should be mentioned in the conclusion of the letter.

Interestingly, the panels considered that it was important to share information about resuscitation plans in both the referral and specialist letters. Recent studies have indicated that timely discussion of preferences for end-of-life care is important to improve quality of life and care in this phase [16]. Our findings are in line with earlier research that doctors take these discussions seriously and need to have clear information to do this effectively [17]. Although outside the scope of this study, it would be interesting to elaborate on the responsibilities in this matter as perceived by GPs, oncology specialists and patients.

Concerning the content of specialists’ letters, the most striking requirement was for the inclusion of detailed information, including the rationale, about diagnosis and treatment. Earlier research by our group showed that detailed information about diagnosis is often available in correspondence, but that key information about possible treatment options and the justification is often lacking [14]. Similarly, it is typically the case that no explicit information is given about whether the treatment is being done with palliative or curative intent [14]. This is important because GPs are not only involved in providing care for patients during and after cancer treatment [6, 7] but also because they are expected to be formally involved in that care [9]. After a patient has visited an oncology specialist, the GP is able to answer questions that arise and to discuss the diagnosis and the treatment options after the patient has digested the initial information. In this way, GPs can bridge the gap between the patient and specialist by delivering care close to home, potentially allaying patient distress and possibly even reducing the burden on secondary care.

In this study, we also asked the panels to indicate their preferences regarding the use of undefined abbreviations in correspondence. We previously reported that abbreviations are common in medical practice (e.g. “BP” for “blood pressure” or “abd.” for “abdomen”) [14]. However, some medical abbreviations can have different meanings depending on the context and specialism (e.g. “OAC” for both “oral anti-conceptive” and “oral anti-coagulant”) and may cause confusion [18]. To ensure mutual understanding, abbreviations should be avoided, even if their meaning is customary and obvious to the writer.

Although the study was performed in the Netherlands, we think that the results may also be of interest to doctors in other healthcare systems. The care for cancer patients often involves several doctors and it is important for them to share all important information. Besides, our results have the potential to inform medical training; they can be used to develop guidelines and they can help develop formats for letters generated from electronic patient files.

A major strength of this study is that it provides important information that can be used by all doctors to improve communication. Another strength is the quality of the item list, which had large content validity because it combined data from a previous study and an extensive literature search, and because participants were asked to suggest additional items. To ensure comprehensibility of the item list, all items were discussed in several meetings, the list was assessed among GPs and oncology specialists and explanations of all items were provided in writing to participants from the patient panel.

The panel quality was key to producing valid outcomes from this study, so we composed each panel with great care by purposive sampling. This was both a strength and a limitation. On the one hand, participants were recruited from across the Netherlands and sampled based on a list of desired characteristics, with the experts showing a high level of consensus, indicating a high probability that the study results could be generalised to cancer care in the Netherlands and in countries with comparable healthcare systems. On the other hand, it is possible that our cohort was not representative of all healthcare providers, given the response rates of 42% and 41% respectively for GPs and medical specialists.

This study is the first to use an iterative approach to identify what information is perceived as relevant and what is perceived as redundant in communication between primary and secondary care. Given that administration, including writing letters, is a time-consuming task for doctors [19], it is important that the focus is on providing only that information considered relevant to the recipient. Such an approach can enhance the effectiveness of communication, lessen administrative burdens and potentially result in cost-saving.

### Conclusion

Our research focused on communication between primary and secondary care before and during the initial treatment of cancer in the Netherlands. Using a consensus approach with panels of GPs, oncology specialists and patients, we obtained a set of items that were deemed relevant for inclusion in referral letters and specialist letters by all groups. The recommendations of this study can be used to develop guidelines for the correct composition of these letters and could be incorporated in computerised systems in both primary and secondary care that generate letters from electronic patient records.

## Electronic supplementary material

ESM 1 (DOCX 202 kb)

## References

[1] Ferlay J, Colombet M, Soerjomataram I, Dyba T, Randi G, Bettio M, Gavin A, Visser O, Bray F (2018). Cancer incidence and mortality patterns in Europe: estimates for 40 countries and 25 major cancers in 2018. Eur J Cancer Published Online First.

[2] Torre LA, Bray F, Siegel RL (2015). Global cancer statistics, 2012. CA Cancer J Clin.

[3] Sussman J, Baldwin LM (2010). The interface of primary and oncology specialty care: from diagnosis through primary treatment. J Natl Cancer Inst Monogr.

[4] Lafferty J, Rankin F, Duffy C, Kearney P, Doherty E, McMenamin M, Coates V (2011). Continuity of care for women with breast cancer: a survey of the views and experiences of patients, carers and health care professionals. Eur J Oncol Nurs.

[5] Nazareth I, Jones L, Irving A, Aslett H, Ramsay A, Richardson A, Tookman A, Mason C, King M (2008). Perceived concepts of continuity of care in people with colorectal and breast cancer - a qualitative case study analysis. Eur J Cancer Care (Engl).

[6] Brandenbarg D, Roorda C, Groenhof F, Havenga K, Berger MY, de Bock GH, Berendsen AJ (2014). Increased primary health care use in the first year after colorectal cancer diagnosis. Scand J Prim Health Care.

[7] Roorda C, de Bock GH, van der Veen WJ, Lindeman A, Jansen L, van der Meer K (2012). Role of the general practitioner during the active breast cancer treatment phase: an analysis of health care use. Support Care Cancer.

[8] Haggerty JL, Reid RJ, Freeman GK, Starfield BH, Adair CE, McKendry R (2003). Continuity of care: a multidisciplinary review. BMJ.

[9] Nederlands Huisartsen Genootschap (2014) Website: Oncologische zorg in de huisartsenpraktijk NHG-standpunt. https://www.nhg.org/themas/publicaties/

[10] Berendsen AJ, Kuiken A, Benneker WHGM, Meyboom-de Jong B, Voorn TB, Schuling J (2009) How do general practitioners and specialists value their mutual communication? A survey. BMC Health Serv Res 9:143. 10.1186/1472-6963-9-143 10.1186/1472-6963-9-143 PMC2736936 19664238

[11] Cabana MD, Jee SH (2004). Does continuity of care improve patient outcomes?. J Fam Pract.

[12] Vermeir P, Vandijck D, Degroote S, Peleman R, Verhaeghe R, Mortier E, Hallaert G, van Daele S, Buylaert W, Vogelaers D (2015). Communication in healthcare: a narrative review of the literature and practical recommendations. Int J Clin Pract.

[13] Hasson F, Keeney S, McKenna H (2000). Research guidelines for the Delphi survey technique. J Adv Nurs.

[14] Stegmann Mariken E., Meijer Jiska M., Nuver Janine, Havenga Klaas, Hiltermann Thijo J.N., Maduro John H., Schuling Jan, Berendsen Annette J. (2018). Correspondence between primary and secondary care about patients with cancer: A qualitative mixed-methods analysis. European Journal of Cancer Care.

[15] Geerse O.P., Wynia K., Kruijer M., Schotsman M.J., Hiltermann T.J.N., Berendsen A.J. (2016). Health-related problems in adult cancer survivors: development and validation of the Cancer Survivor Core Set. Supportive Care in Cancer.

[16] Brinkman-Stoppelenburg A, Rietjens JAC, Van Der Heide A (2014). The effects of advance care planning on end-of-life care: a systematic review. Palliat Med.

[17] Oosterink John J, Oosterveld-Vlug Mariska G, Glaudemans Jolien J, Pasman H Roeline W, Willems Dick L, Onwuteaka-Philipsen Bregje D (2016). Interprofessional communication between oncologic specialists and general practitioners on end-of-life issues needs improvement. Family Practice.

[18] Girbes AC, Girbes ARJ (2017) Abbreviations in daily language: stop it. (Article in Dutch). Ned Tijdschr Geneeskd. 161:D1414 28612696

[19] Rao SK, Kimball AB, Lehrhoff SR, Hidrue MK, Colton DG, Ferris TG, Torchiana DF (2017). The impact of administrative burden on academic physicians: results of a hospital-wide physician survey. Acad Med.

