# Supplementary material for: Correspondence between primary and secondary care about patients with cancer: a Delphi consensus study
Source: Support Care Cancer. 2019 Mar 1;27(11):4199–205. doi: 10.1007/s00520-019-04712-5 (PMC6803614; doi:10.1007/s00520-019-04712-5)
Supplement: Supplementary file 1 — (DOCX 202 kb) [file 520_2019_4712_MOESM1_ESM.docx]

**Supplement 1: Literature search**

| **We used four different MeSH and key term strings covering primary care, secondary care, written information exchange and grading parameter (quality, opinion).**  **Search strategy MEDLINE:**  ((((Primary Care[Title/Abstract] OR GP[Title/Abstract] OR primary health care[MeSH Terms] OR family practice[MeSH Terms] OR family practice[Title/Abstract] OR family physician[Title/Abstract] OR family physicians[Title/Abstract] OR general practitioners[MeSH Terms] OR general practitioner[Title/Abstract] OR general practitioners[Title/Abstract] OR primary[Title/Abstract] OR general practice[MeSH Terms] OR general practice[Title/Abstract]))  AND  (secondary care[MeSH Terms] OR hospital-based[Title/Abstract] OR hospital care[Title/Abstract] OR secondary care centers[MeSH Terms] OR secondary care[Title/Abstract] OR specialist*[Title/Abstract] OR specialized[Title/Abstract] OR specialised[Title/Abstract] OR consultants[MeSH Terms] OR consultant*[Title/Abstract])) AND  (correspondence as topic[MeSH Terms] OR correspondence[Title/Abstract] OR written communication[Title/Abstract] OR writing[Title/Abstract] OR patient discharge summaries[MeSH Terms] OR health information exchange[MeSH Terms] OR information exchange[Title/Abstract] OR discharge summaries[Title/Abstract] OR discharge summary[Title/Abstract] OR information transfer[Title/Abstract] OR letter*[Title/Abstract]))  AND  (Quality[Title/Abstract] OR content[Title/Abstract] OR deficit*[Title/Abstract] OR deficienc*[Title/Abstract] OR Value[Title/Abstract] OR contain[Title/Abstract] OR view[Title/Abstract] OR perspective*[Title/Abstract] OR opinion*[Title/Abstract] OR assessment[Title/Abstract])  AND  (Journal Article[ptyp] AND full text[sb] AND ("1990/01/01"[PDat] : "3000/12/31"[PDat]) AND English[lang])  **Search strategy Web Of Science:**  TOPIC: ("Primary Care" OR "GP" OR "primary health care" OR "family practice" OR "family physician" OR "family physicians" OR "general practitioner" OR "general practitioners" OR "primary" OR "general practice")  AND  TOPIC: ("secondary care" OR "hospital-based" OR "hospital care" OR "specialist" OR "specialists" OR "specialized" OR "specialised" OR "consultant" OR "consultants")  AND  TOPIC: ("correspondence" OR "written communication" OR "writing" OR "information exchange" OR "discharge summaries" OR "discharge summary" OR "information transfer" OR "letter" OR "letters")  AND TOPIC: ("Quality" OR "content" OR "deficit" OR "deficits" OR "deficiency" OR "deficiencies" OR "Value" OR "contain" OR "view" OR "views" OR "perspective" OR "perspectives" OR "opinion" OR "opinions" OR "assessment")  Refined by: DOCUMENT TYPES: (ARTICLE OR REVIEW) AND LANGUAGES: (ENGLISH)  Timespan: 1990-2016. Indexes: SCI-EXPANDED, SSCI, A&HCI, ESCI.  The following inclusion criteria were defined:   - original research, systematic-review or meta-analysis; - published after 1990 in peer-reviewed journal; - English language; - concerned medical correspondence in general or correspondence in cancer care. |
| --- |

**Figure S1a:** Search strategy

**Figure S1b:** Flow diagram for the literature search

**Figure S1c:** Flow diagram for composing the original item list for panel review

**Supplement 2:** Complete item list and results of both Delphi rounds

| Category | Item | Round one (median score) | | | | Round two (agreement %)* | | | | Status |
| --- | --- | --- | --- | --- | --- | --- | --- | --- | --- | --- |
| Referral letter | | **Overall** | GPs | MSs | Ps | **Overall** | GPs | MSs | Ps |  |
| Background | Reason for referral/referral question  New referral or re-referral  Level of urgency  Reanimation policy  Active diagnoses/relevant past medical history  Other/miscellaneous past medical history  Smoking habits  Number of packyears  Alcohol habits  Number of alcoholic intakes per day  Soft/hard drug habits  Frequency and dosage of drugs  Diet habits  Statement of level of involvement of the patient with the referral  Expectations of the patient regarding the referral  Expectations of family regarding the referral  Information about desired level of involvement of the patient in the care process | **4**  **3**  **4**  **4**  **4**  **3**  **3**  **3**  **3**  **3**  **3**  **3**  **2**  **2**  **3**  **2**  **3** | 4  3  3.5  4  4  3  3  3  3  2  3  3  2.5  2.5  3  2  3 | 4  3  4  4  4  4  3  3  3  3  3  3  2  2  3  2  3 | 4  3  4  4  4  3  3  3  3  3  3  3  3  3  3  2  4 | **99**  **82**  **87**  **54**  **97**  **67**  **75**  **88**  **74**  **83**  **72**  **83**  **-**  **-**  **78**  **-**  **78** | 100  82  82  57  100  86  64  89  61  79  64  82  71  79 | 100  85  91  55  100  49  76  85  79  85  76  82  79  94 | 93  73  87  47  87  73  93  93  87  87  80  87  87  40 | Included  Excluded R2  Included  Included  Included  Excluded R2  Excluded R2  Excluded R2  Excluded R2  Excluded R2  Excluded R2  Excluded R2  Excluded R1  Excluded R1  Excluded R2  Excluded R1  Excluded R2 |
| Allergies and medication | Medication allergies  Non-medication allergies  Symptoms during allergic reaction  List of active medication  Dose and frequency of active medication | **4**  **3**  **3**  **4**  **4** | 4  3  3  4  4 | 4  4  4  4  4 | 4  4  4  3  3 | **90**  **72**  **82**  **84**  **74** | 96  68  79  100  89 | 88  76  88  88  70 | 80  73  73  47  53 | Included  Excluded R2  Excluded R2  Included  Included |
| History and symptoms | Presenting symptoms  History of symptoms  Symptoms in other tract  Psychological wellbeing  Oncological family history  Non-oncological family history | **4**  **4**  **2**  **3**  **3**  **2** | 4  3.5  3  3  3  2 | 4  4  2  3  3  2 | 4  4  3  3  3  2 | **95**  **75**  **-**  **63**  **70**  **-** | 96  82  71  64 | 94  70  70  82 | 93  73  33  53 | Included  Included  Excluded R1  Excluded R2  Excluded R2  Excluded R1 |
| Psychosocial information | Living conditions  Home care  Mobility (e.g. car/use of public traffic services)  Hobbies  Employment  Impact of disease and treatment on employment  Impact of disease and treatment on direct environment  Understanding/awareness of illness  Coping and coping habits  Level of social support  Level of adherence to therapy  Religious and cultural beliefs about disease and death  Need for an interpreter  Complementary healthcare methods used by this patient | **3**  **3**  **2**  **2**  **2**  **3**  **2**  **3**  **3**  **3**  **3**  **3**  **4**  **2** | 3  3  2  2  2  3  3  3  3  3  3  2  4  2 | 3  3  2  2  2  2  2  3  3  3  3  3  4  3 | 2  3  3  2  2  3  2  3  3  3  3  3  3  2 | **84**  **84**  **-**  **-**  **-**  **92**  **-**  **78**  **83**  **86**  **76**  **84**  **70**  **-** | 86  89  96  79  82  86  75  93  61 | 85  88  91  79  85  91  85  88  85 | 80  67  87  73  80  73  60  60  53 | Excluded R2  Excluded R2  Excluded R1  Excluded R1  Excluded R1  Excluded R2  Excluded R1  Excluded R2  Excluded R2  Excluded R2  Excluded R2  Excluded R2  Included  Excluded R1 |
| Physical examination | First impression (e.g. healthy or really ill)  Abnormal findings, relevant for the current problem  Normal findings, relevant for the current problem  Abnormal findings, not relevant for the current problem  Normal findings, not relevant for the current problem  Performance status (level of functioning 0-100%) | **3**  **4**  **3**  **2**  **2**  **3** | 3  4  3  3  2  2 | 3  3  3  2  2  3 | 3  4  3  3  2  3 | **76**  **91**  **76**  **-**  **-**  **72** | 79  100  86  89 | 79  85  79  61 | 67  87  53  67 | Excluded R2  Included  Excluded R2  Excluded R1  Excluded R1  Excluded R2 |
| Investigations | Abnormal findings, relevant for the current problem  Normal findings, relevant for the current problem  Abnormal findings, not relevant for the current problem  Normal findings, not relevant for the current problem  Upcoming results (investigation already finished) | **4**  **3**  **3**  **2**  **3** | 4  3  3  2  3 | 4  3  2  2  3 | 4  3  3  2  4 | **95**  **78**  **74**  **-**  **67** | 100  75  71  54 | 97  91  76  76 | 80  53  73  73 | Included  Excluded R2  Excluded R2  Excluded R1  Excluded R2 |
| Policy and treatment | Diagnosis/provisional diagnosis  Other possible diagnoses  Argumentation for chosen treatment  Used treatments, with effect  Used treatments, without effect  Reaction to former treatments  Factors influencing treatment effect  Ideas from the GP regarding what could be a good treatment  Ideas from the GP regarding his/her own role in the future care for this disease  Lifestyle advices given to the patient | **3**  **3**  **3**  **3**  **3**  **3**  **3**  **3**  **3**  **3** | 4  3  3  3  3  3  3  3  3  3 | 3  3  3  3  3  3  4  3  3  3 | 4  4  3  3  3  3  4  3  4  3 | **51**  **84**  **86**  **50**  **61**  **55**  **55**  **81**  **67**  **87** | 32  79  86  61  68  64  50  89  71  82 | 61  88  91  52  58  58  64  79  7  91 | 67  87  73  27  53  33  47  67  33  87 | Excluded R2  Excluded R2  Excluded R2  Excluded R2  Excluded R2  Excluded R2  Excluded R2  Excluded R2  Excluded R2  Excluded R2 |
| Summary and closure | Summary/conclusion  What is told to the patient  Reaction from the patient regarding the given information  Involvement of other medical doctors  Care that is arranged for at home  Scientific studies patient is eligible for  Scientific studies patient is participating in  Is the medical specialist called before sending the letter  Has the patient received a copy of the letter | **3**  **3**  **3**  **3**  **3**  **1**  **3**  **3**  **2** | 4  3  3  3  3  1  3  3  2 | 3  3  3  3  3  1  3  2  2 | 4  4  3  3  2  2  3  3  3 | **47**  **63**  **82**  **59**  **90**  **-**  **58**  **76**  **-** | 61  64  86  50  93  64  75 | 42  67  88  67  94  58  79 | 33  53  60  60  73  47  73 | Excluded R2  Excluded R2  Excluded R2  Excluded R2  Excluded R2  Excluded R1  Excluded R2  Excluded R2  Excluded R1 |
| Specialist letter | |  |  |  |  |  |  |  |  |  |
| Background | Reason/purpose of the letter  Reanimation policy  Active diagnoses/relevant past medical history  Other/miscellaneous past medical history  Smoking habits  Number of packyears  Alcohol habits  Number of alcoholic intakes per day  Soft/hard drug habits  Frequency and dosage of drugs  Diet habits  Information about desired level of involvement of the patient in the care process | **4**  **4**  **4**  **3**  **3**  **2**  **2**  **2**  **3**  **3**  **2**  **3** | 3  4  4  2.5  3  2  2  2  3  3  3  3 | 4  4  4  4  3  3  3  3  3  3  3  3 | 3  4  4  2  2  2  2  2  2  2  2  4 | **96**  **75**  **96**  **73**  **81**  **-**  **-**  **-**  **80**  **85**  **-**  **75** | 92  79  89  92  97  93  96  86 | 100  81  100  60  72  78  84  81 | 93  53  100  67  73  60  67  40 | Included  Included  Included  Excluded R2  Excluded R2  Excluded R1  Excluded R1  Excluded R1  Excluded R2  Excluded R2  Excluded R1  Excluded R2 |
| Allergies and medication | Medication allergies  Non-medication allergies  Symptoms during allergic reaction  List of active medication  Dose and frequency of active medication | **4**  **3**  **3**  **4**  **4** | 4  3  3  4  4 | 4  3  3  4  4 | 3  3  3  3  4 | **90**  **77**  **76**  **92**  **89** | 89  86  86  96  93 | 94  72  72  97  84 | 80  73  67  73  73 | Included  Excluded R2  Excluded R2  Included  Included |
| History and symptoms | Presenting symptoms  History of symptoms  Symptoms in other tract  Psychological wellbeing  Oncological family history  Non-oncological family history | **4**  **3**  **2**  **3**  **3**  **2** | 3  3  2.5  3  3  2 | 4  4  2  3  3  2 | 4  3  2  3  3  2 | **95**  **75**  **-**  **71**  **64**  **-** | 89  82  71  75 | 100  72  81  63 | 93  67  47  47 | Included  Excluded R2  Excluded R1  Excluded R2  Excluded R2  Excluded R1 |
| Psychosocial information | Living conditions  Home care  Mobility (e.g. car/use of public traffic services)  Hobbies  Employment  Impact of disease and treatment on employment  Impact of disease and treatment on direct environment  Understanding/awareness of illness  Coping and coping habits  Level of social support  Level of adherence to therapy  Religious and cultural beliefs about disease and death  Need for an interpreter  Complementary healthcare methods used by this patient | **2**  **2**  **2**  **2**  **2**  **2**  **2**  **3**  **3**  **2**  **3**  **2**  **2**  **2** | 2  3  2  2  2  2.5  2  3  3  2  3  2  2  2 | 2  2  2  2  2  2  2  3  2  2  3  2  2  2 | 2  2  2  1  2  2  2  3  3  2  3  3  3  2 | **-**  **-**  **-**  **-**  **-**  **-**  **-**  **73**  **81**  **-**  **75**  **-**  **-**  **-** | 75  89  82 | 78  81  75 | 60  67  60 | Excluded R1  Excluded R1  Excluded R1  Excluded R1  Excluded R1  Excluded R1  Excluded R1  Excluded R2  Excluded R2  Excluded R1  Excluded R2  Excluded R1  Excluded R1  Excluded R1 |
| Physical examination | First impression (e.g. healthy or really ill)  Abnormal findings, relevant for the current problem  Normal findings, relevant for the current problem  Abnormal findings, not relevant for the current problem  Normal findings, not relevant for the current problem  Performance status (level of functioning 0-100%) | **3**  **4**  **3**  **3**  **2**  **3** | 3  4  2  3  2  2.5 | 4  4  3  3  2  4 | 3  4  3  2  3  3 | **65**  **93**  **72**  **63**  **-**  **61** | 82  96  86  71  89 | 59  91  69  66  41 | 47  93  53  40  53 | Excluded R2  Included  Excluded R2  Excluded R2  Excluded R1  Excluded R2 |
| Investigations | Abnormal findings, relevant for the current problem  Normal findings, relevant for the current problem  Abnormal findings, not relevant for the current problem  Normal findings, not relevant for the current problem  Upcoming results (investigation already finished) | **4**  **3**  **3**  **2**  **3** | 4  3  3  2  3 | 4  3  3  2  3 | 4  3  3  3  4 | **99**  **76**  **57**  **-**  **61** | 100  86  61  57 | 97  72  56  69 | 100  67  40  53 | Included  Excluded R2  Excluded R2  Excluded R1  Excluded R2 |
| Diagnosis | Diagnosis/provisional diagnosis  Other possible diagnoses  TNM classification  Whether the tumour is localized of metastasized.  Size and local extension of the primary tumour (T)**  Level of metastases tot regional lymph nodes (N)  Description of metastases (M)  Results of intercollegiate consultations  Summary/conclusion of multidisciplinary tumour board meeting  Full report of multidisciplinary tumour board meeting  Actions/treatments done by other medical specialists | **4**  **3**  **3**  **4**  **3**  **4**  **4**  **3**  **4**  **2**  **3** | 4  3  3  4  3  3.5  4  3  4  2  3 | 4  3  4  4  3  3  3  3  4  2  3 | 4  4  4  4  4  4  4  4  4  2  4 | **99**  **55**  **37**  **100**  **37****  **95**  **93**  **43**  **96**  **-**  **76** | 100  57  32  100  29  93  96  50  100  86 | 100  53  31  100  44  94  100  44  91  75 | 93  53  60  100  60  100  73  27  100  60 | Included  Excluded R2  Excluded R2  Included  Included  Included  Included  Excluded R2  Included  Excluded R1  Excluded R2 |
| Policy and treatment | Possible treatment options  Chosen treatment (e.g. watchful waiting, surgery)  Argument for chosen treatment  Alternative treatment options in case of failure of first choice  Whether the intent of treatment is curative of palliative  Prognosis with treatment  Prognosis without treatment  Former treatments with positive effects  Former treatments without positive effects  Response on former treatments  Lifestyle advices given to the patient  Treatment schedule chemotherapy  Treatment schedule radiotherapy  Short-term side-effects**  Treatment recommendations for short-term side-effects  Expected course of the disease  Treatment recommendations for future symptoms of the disease  Nutritional advices given to the patient  Psychosocial care agreed on  Needed care at home | **4**  **4**  **4**  **3**  **4**  **4**  **3**  **4**  **3**  **4**  **3**  **3**  **3**  **3**  **3**  **3**  **3**  **3**  **3**  **3** | 3  4  4  3  4  3.5  3  4  3  3.5  3  3  3  3  3  3  3  2.5  3  3 | 4  4  4  3  4  3  3  4  4  4  3  3  3  3  3  3  3  3  3  3 | 4  4  3  3  4  4  4  4  4  4  3  3  3  3  3  4  4  4  3  3 | **93**  **100**  **95**  **73**  **99**  **85**  **51**  **96**  **53**  **96**  **61**  **44**  **47**  **24**  **39**  **57**  **59**  **72**  **61**  **72** | 100  100  100  64  100  93  43  100  46  100  75  46  46  25  32  57  61  75  64  71 | 88  100  94  91  100  78  72  91  59  91  56  41  47  25  53  72  75  81  72  78 | 93  100  87  53  93  87  20  100  53  100  73  47  47  20  20  27  20  47  33  60 | Included  Included  Included  Excluded R2  Included  Included  Excluded R2  Included  Excluded R2  Included  Excluded R2  Excluded R2  Excluded R2  Included  Excluded R2  Excluded R2  Excluded R2  Excluded R2  Excluded R2  Excluded R2 |
| Hospitalization | Discharge destination  Course (what happened) during hospitalization  Complications  Summary/conclusion of surgical procedure  Full report of surgical procedure  Medication changes  Actual list of medication  Active medical problems at discharge  Care that is arranged for at home  Intentions regarding residual medical materials (e.g., drains or stitches) | **4**  **3**  **4**  **3**  **2**  **4**  **4**  **4**  **3**  **4** | 4  3  4  3  2  4  4  4  3  4 | 4  4  4  3  2  4  4  4  3  4 | 3  3  3  3  2  4  3  4  3  4 | **85**  **48**  **95**  **41**  **-**  **89**  **89**  **99**  **51**  **96** | 93  50  96  46  96  89  100  50  100 | 84  41  97  41  88  91  97  50  100 | 73  60  87  33  80  87  100  53  80 | Included  Excluded R2  Included  Excluded R2  Excluded R1  Included  Included  Included  Excluded R2  Included |
| Summary and closure | Summary/conclusion  What the patient has been told  Reaction from the patient regarding the given information  Involvement of other medical doctors  Scientific studies patient is eligible for  Scientific studies patient is participating in**  Is the medical specialist called before sending the letter  Has the patient received a copy of the letter?  For what problems should the patient contact  the medical specialist?  Whom the patient should contact  With whom (which specialism) has the patient appointments during the  upcoming period?  Frequency of appointments  Date and time of appointments  Request to the GP  Requests to other medical specialists | **4**  **4**  **3**  **3**  **3**  **3**  **3**  **2**  **4**  **4**  **3**  **2**  **2**  **4**  **3** | 4  4  3  3  3  3  3  2.5  4  4  3  2  2  4  3 | 4  4  3  3  3  4  3  2  3  3  3  2  3  4  3 | 4  4  4  4  3  4  3  3  4  4  3  3  2  3  3 | **100**  **95**  **69**  **51**  **80**  **21****  **80**  **-**  **87**  **85**  **60**  **-**  **-**  **95**  **67** | 100  100  68  50  89  14  75  93  96  61  96  68 | 100  91  72  59  81  16  88  78  72  63  97  63 | 100  93  67  33  60  47  73  93  93  53  87  73 | Included  Included  Excluded R2  Excluded R2  Excluded R2  Excluded R2  Excluded R2  Excluded R1  Included  Included  Excluded R2  Excluded R1  Excluded R1  Included  Excluded R2 |
| Closure of treatment | Known late effects of treatment  Given cumulative dose of a treatment  Follow-up procedure, which specialism  Follow-up procedure, frequency | **3**  **2**  **3**  **3** | 3  2  3  3 | 3  2  3  3 | 4  3  3  3 | **51**  **-**  **43**  **60** | 50  54  68 | 63  34  63 | 27  40  40 | Excluded R2  Excluded R1  Excluded R2  Excluded R2 |

*: items with overall median scores of four: percentage agreed with including, for item with overall median scores of three: percentage agreed with excluding. **: items with overall median scores of three but included on the ultimate consensus list after round two. - : item excluded after round one, so agreement round 2 is not applicable.

R1: after Round 1; R2: after Round 2.
